# Mental Health and Coping Strategies of Health Communicators Who Faced Online Abuse During the COVID-19 Pandemic: Mixed Methods Study

**DOI:** 10.2196/68483

**Published:** 2025-04-02

**Authors:** Lisa Wight, Chris Tenove, Saima Hirani, Heidi Tworek

**Affiliations:** 1 Centre for the Study of Democratic Institutions School of Public Policy and Global Affairs University of British Columbia Vancouver, BC Canada; 2 School of Nursing University of British Columbia Vancouver, BC Canada

**Keywords:** mental health, online harassment, online abuse, coping strategies, resilience, social media, online advocacy, public health communication, health communication

## Abstract

**Background:**

During the COVID-19 pandemic, health experts used social media platforms to share information and advocate for policies. Many of them faced online abuse, which some reported took a toll on their mental health and well-being. Variation in the impacts of online abuse on mental health, well-being, and professional efficacy suggest that health communicators may differ in their coping strategies and ultimately their resilience to such abuse.

**Objective:**

We aimed to explore the impacts of online abuse on health communicators’ mental health and well-being as well as their emotion- and problem-focused coping strategies.

**Methods:**

We recruited health communicators (public health officials, medical practitioners, and university-based researchers) in Canada who engaged in professional online communication during the COVID-19 pandemic. In phase 1, semistructured interviews were conducted with 35 health communicators. In phase 2, online questionnaires were completed by 34 individuals before participating in workshops. Purposive recruitment resulted in significant inclusion of those who self-identified as racialized or women. Interview and workshop data were subjected to inductive and deductive coding techniques to generate themes. Descriptive statistics were calculated for selected questionnaire questions.

**Results:**

In total, 94% (33/35) of interviewees and 82% (28/34) of questionnaire respondents reported experiencing online abuse during the study period (2020-2022). Most health communicators mentioned facing an emotional and psychological toll, including symptoms of depression and anxiety. Racialized and women health communicators faced abuse that emphasized their ethnicity, gender identity, and physical appearance. Health communicators’ most common emotion-focused coping strategies were withdrawing from social media platforms, avoiding social media platforms altogether, and accepting online abuse as unavoidable. Common problem-focused coping strategies included blocking or unfriending hostile accounts, changing online behavior, formal help-seeking, and seeking peer support. Due to the impacts of online abuse on participants’ mental health and well-being, 41% (14/34) of the questionnaire respondents seriously contemplated quitting health communication, while 53% (18/34) reduced or suspended their online presence. Our findings suggest that health communicators who used problem-focused coping strategies were more likely to remain active online, demonstrating significant professional resilience.

**Conclusions:**

Although health communicators in our study implemented various emotion- and problem-focused coping strategies, they still faced challenges in dealing with the impacts of online abuse. Our findings reveal the limitations of individual coping strategies, suggesting the need for effective formal organizational policies to support those who receive online abuse and to sanction those who perpetrate it. Organizational policies could improve long-term outcomes for health communicators’ mental health and well-being by mitigating online abuse and supporting its targets. Such policies would bolster professional resilience, ensuring that important health information can still reach the public and is not silenced by online abuse. More research is needed to determine whether gender, race, or other factors shape coping strategies and their effectiveness.

## Introduction

### Background

From the start of the COVID-19 pandemic, public health officials, medical practitioners, university-based researchers, health journalists, and other health experts played a crucial role in shaping public opinions and behaviors. To inform publics and counter poor-quality information, many health experts increased their use of social media platforms: frontline health care workers creating TikTok videos [1], medical professionals countering misinformation on Twitter [2], and physicians and researchers posting on Facebook, Instagram, and Twitter [3,4]. These health communicators could be considered an emergent community of practice, meaning they encountered many similar opportunities and challenges of engaging audiences through social media platforms.

Many health communicators were exposed to online harassment and abuse, ranging from trivial criticisms to sexual harassment and violent threats [5,6]. This abuse was faced by those communicating as individuals or on behalf of institutions. Health communicators were typically unprepared for the abuse that often follows online advocacy [7], which was exacerbated by a lack of existing institutional protections and supports [7,8].

### Exposure to Online Abuse

In a survey conducted from February 2019 to March 2019, 23% of 464 physicians in the United States reported being personally attacked on social media, primarily for advocacy on topics such as vaccines, gun control, and abortion [9]. Research suggests that the online harassment experienced by health communicators worsened during the COVID-19 pandemic [10,11]. In a 2022 survey of 359 physicians, biomedical scientists, and trainees in the United States, 228 (64%) reported harassment on social media related to comments they had made about the COVID-19 pandemic [12]. Similarly, more than two-thirds of 321 scientists responding to a *Nature* survey in 2021, predominantly located in the United States, the United Kingdom, and Germany, reported negative experiences because of their media interviews or social media comments regarding COVID-19 [10]. In total, 22% of these respondents had received threats of physical or sexual violence, and 15% had received death threats [10]. Threats of violence illustrate that online abuse is not merely confined to the internet [13]. Escalating violence against health care workers in Canada during the COVID-19 pandemic prompted the Canadian Medical Association to call for legislation in 2021 that would protect health care workers from aggressive patients and protesters, both online *and* in-person [14].

Forms of online abuse may differ with individuals’ gender identity, race or ethnicity, professional role, and other factors. For instance, women health communicators and journalists have faced more gendered or sexualized abuse than men [6,9,15].

### Impacts of Online Abuse

Research suggests that online abuse can have serious negative consequences for health communicators’ mental health and well-being: individuals who have experienced online harassment consistently report emotional distress and fear [2,9]. In a prepandemic survey, 62.4% (63/101) of prominent medical science communicators reported some negative mental health impacts, including depression, anxiety, and stress because of public engagement [6]. While most mental health impacts they reported were minor, 15% of them reported considerable or significant mental health ramifications [6]. Mental health consequences of online abuse can be disproportionately experienced due to gender, race or ethnicity, and other sociodemographic factors [15]. Throughout the COVID-19 pandemic, many health communicators have spoken out about the toll that negative comments and personal attacks have taken on their mental health [5,11]. In the *Nature* survey, more than 40% of 321 scientists reported experiencing emotional or psychological distress after commenting about the COVID-19 pandemic in traditional media interviews or on social media [10].

Some health communicators have expressed a desire to reduce or stop their online advocacy [6,10,12]. Thus, online abuse and its mental health consequences may undermine individuals’ professional capacity, reducing the amount of engagement within the communication sector. If certain voices (eg, women and racialized individuals) are pushed out [16], then the diversity of perspectives within this sector will be in jeopardy.

### Coping and Other Responses to Online Abuse

Variation in the impacts of online abuse on mental health, well-being, and professional efficacy suggests that health communicators differed in their coping strategies and ultimately their resilience to such abuse. A person who has experienced harassment will tend to adopt one or more strategies to “cope with it” [16]. Lazarus and Folkman [17] developed a seminal model to understand coping as either emotion- or problem-focused. Emotion-focused coping strategies help regulate emotional responses to a stressful situation, whereas problem-focused coping strategies aim to manage or alter the situation itself [17]. The effectiveness of emotion- and problem-focused coping strategies has been debated, but those who rely predominantly on emotion-focused coping strategies report significant negative emotions and poor mental health outcomes, such as depression [18].

Research on the coping strategies used by individuals who receive online abuse has primarily been conducted with journalists, scholars, and students [16,18-20], rather than health communicators. Many studies have further stratified emotion- and problem-focused coping strategies. For example, Scarduzio et al [21] put forth 11 “types” of emotion-focused strategies (eg, ignoring negative comments) and 5 “types” of problem-focused strategies (eg, blocking hostile users) used by university students in response to online sexual harassment. In this study, we leveraged the model developed by Lazarus and Folkman as well as the examples of digital coping strategies given by Scarduzio et al to examine how health communicators responded to online abuse.

### Objectives

Despite the broad adoption of social media by health care providers, scientists, and public health officials and the increasing recognition of online abuse they have received, no study has focused on the mental health consequences of *online* harassment for health communicators during the COVID-19 pandemic. Thus, our study aimed to explore (1) the impacts of online abuse on health communicators’ mental health and well-being during the COVID-19 pandemic and (2) the emotion- and problem-focused coping strategies health communicators used to manage online abuse.

## Methods

### Study Design

This study is part of a larger participatory action research project on an emergent community of practice of health communicators. Relationships with health communicators were developed through direct outreach and through a partnership with ScienceUpFirst, a Canadian initiative that works with science and health experts to address misinformation. We used mixed methods, including semistructured interviews, an online questionnaire, and 2 workshops to examine the impacts of online abuse on Canadian health communicators during the COVID-19 pandemic.

### Participant Recruitment

Health communicators for the larger participatory action research project were purposively recruited in 2 phases, emphasizing significant inclusion of those who self-identified as women or racialized. We use the term “racialized” rather than “visible minority groups” or the terms “Black,” “Indigenous,” and “people of color,” following recommendations to use the former term by the Canadian government [22] and academics [23], arguing that race “does not exist as a biological concept to distinguish between human beings, but that social processes of racialization are inherently linked to major forms of historical, social, economic, and cultural oppression, including slavery and colonialism” [23].

During the first phase, research team members monitored traditional and social media platforms to identify people in Canada who were actively discussing public health measures online during the COVID-19 pandemic. Team members contacted a subset of these individuals by email, seeking participation from health communicators of diverse gender identities, ethnicities, and professional roles (eg, public health officials, health care professionals, university-based or civil society health experts, and health journalists). Health communicators who replied (N=35) were invited to participate in a virtual one-on-one semistructured interview, after informed consent was obtained.

During the second phase, team members recruited health communicators to participate in 2 workshops. Participants were recruited through #ScienceUpFirst affiliate groups and the authors’ professional networks, seeking similar forms of diversity to the first phase. Journalists were intentionally excluded from the second phase because exploratory conversations with journalists and other health communicators suggested that combining these groups might put participants in awkward professional predicaments and because there has already been extensive research on online abuse of journalists [15,19,24]. In this phase, health communicators (N=34) first completed an online questionnaire to collect data on sociodemographic characteristics, communication activities, and experiences of harassment. Then, they participated in 1 of 2 virtual small-group 2-hour workshops. Observations from these workshops were taken from the research team members’ notes because no audio-recordings or transcripts of the discussions were made.

### Data Collection and Analysis

#### Quantitative Data

Given that there are no validated scales for online abuse of individuals in their professional capacities, we developed a questionnaire by drawing on existing questionnaires [13], including one previously created by the research team members [25] and another created by Ipsos to survey journalists’ experiences of online harm [24]. Alongside the frequency, causes, and sources of online abuse, our questionnaire assessed how participants responded to online harm and how they changed their personal and professional work as health communicators due to online abuse. Simple descriptive statistics were calculated for specific questions using Microsoft Excel. All data were anonymized to protect participant confidentiality and were stored in a secure electronic database.

#### Qualitative Data

One-on-one interviews (N=35) were conducted by a research team member and recorded over Zoom (Zoom Communications, Inc) between December 2021 and June 2023. Each interview lasted between 40 and 90 minutes. A semistructured interview guide was used to conduct the interviews, which allowed researchers to compare participants’ responses to set questions within the guide and explore other insights based on responses. Questions addressed issues, including the form and frequency of online abuse, professional and mental health impacts, and the online and offline actions that individuals took to respond to abuse. Zoom audio-recordings were transcribed verbatim.

For the first round of coding, our principal researcher created an initial list of deductive codes from literature on online harassment of politicians and journalists [16,25]. Three team members then coded approximately 15% (5/35) of the interview transcripts using this list, meeting regularly to discuss whether deductive codes and their definitions should be modified and whether new inductive codes should be added to the list. A revised codebook was created, and the remaining interview transcripts were independently coded using ATLAS.ti (Lumivero) software. Team members continued to discuss coding during this process to ensure their shared fidelity to the revised codebook.

A second round of coding was undertaken to examine interview data about health communicators’ mental health and well-being in more detail. One team member coded all interview excerpts that had been tagged with the “Impact: Mental health” code during the first round of coding. Inductive coding techniques were used to generate themes around mental health and resilience. Deductive coding techniques were used to identify emotion- and problem-focused coping strategies from the model developed by Lazarus and Folkman [17] and the study by Scarduzio et al [21]. Patterns across the excerpts were noted, and our team met 4 times to collectively categorize and interpret themes.

### Ethical Considerations

This study was reviewed and approved by the University of British Columbia Behavioural Research Ethics Board (H21-01503 and #H22-01816). For one-on-one interviews, all participants received a consent statement outlining the study, potential risks and benefits, and measures to protect their personal information. The interviewer reviewed these details at the start of the interview and obtained verbal consent before proceeding. For individuals who completed surveys and participated in workshops, all participants signed a consent form that described the study, potential risks and benefits, and measures to protect their personal information.

Discussing online abuse can be difficult. Both the consent statement and consent form emphasized that participation was entirely voluntary, that individuals could stop participating in the interview or focus group at any time, and that they could withdraw from the study at any point. During interviews, if a participant expressed or displayed discomfort, the interviewer offered to pause, end the interview, or move to another question. For workshops, all participants received a community guidelines document in advance to ensure that group discussions were conducted safely and inclusively.

Data from this project are stored on password-protected hard drives and University of British Columbia–managed cloud storage, accessible only to core research team members registered as part of our research ethics board certification.

## Results

### Participant Characteristics

Table 1 outlines the sociodemographic characteristics of the interviewees (N=35) and questionnaire respondents (N=34). Most participants were living and working in either British Columbia or Ontario, Canada’s 2 most populous English-speaking provinces. More women participated in both phases of this study than men, but both phases had an equal number of self-identified racialized and White participants.

**Table 1:** Sociodemographic characteristics of the participants from individual interviews and questionnaires.

Characteristics			Phase 1: Interviews (N=35), n (%)		Phase 2: Questionnaires (N=34), n (%)
**Self-identified gender**					
	Woman	20 (57)		21 (62)	
	Man	15 (43)		13 (38)	
	Nonbinary	0 (0)		0 (0)	
	Prefer not to answer	0 (0)		0 (0)	
**Self-identified ethnicity**					
	Racialized	17 (49)		17 (50)	
	White	18 (51)		16 (47)	
	Prefer not to answer	0 (0)		1 (3)	
**Province of residence**					
	Alberta	5 (14)		1 (3)	
	British Columbia	15 (43)		11 (32)	
	Ontario	11 (31)		20 (59)	
	Quebec	3 (9)		1 (3)	
	Yukon	1 (3)		0 (0)	
	Other	0 (0)		1 (3)	
**Primary professional role^a^**					
	Public health official^b^	8 (23)		9 (26)	
	Medical professional	10 (29)		9 (26)	
	Health journalist	9 (26)		0 (0)	
	University-based or civil society expert	8 (23)		6 (18)	
	Other^c^	0 (0)		10 (29)	

^a^While several participants belong to more than one category (eg, medical professional *and* university-based expert), we categorized participants based on their *primary* professional role.

^b^Employed by a public health agency, provincial government, or federal government.

^c^Employed by a nonprofit organization, research institute, community health center, science center, or self-employed.

### Impacts of Online Harassment on Health Communicators’ Mental Health and Well-Being

In the first phase, 94% (33/35) of the interviewees reported facing some form of online abuse since the pandemic began. Of the 2 interviewees who had not faced online abuse, one managed a health agency’s general accounts and the other primarily relied on staff to manage her accounts. In the second phase, we asked the participants more detailed questions about the frequency of online harassment they experienced in the last 6 months. In total, 82% (28/34) of the questionnaire respondents received online threats, harassment, or false claims on multiple occasions in the last 6 months.

About 38% (13/34) of the questionnaire respondents claimed that their nationality or ethnic background was the reason they were targeted with online harassment. Racialized interviewees described how the online abuse they encountered differed from their White colleagues. For instance, a racialized woman stated:

There are random people out there that don’t like you just because of who you are. Not necessarily because of what you say... I have female colleagues who are White... who say the same thing and don’t get the reaction that I get.

Civil society expert

These sentiments were repeated by multiple racialized workshop participants.

In total, 18% (6/34) of the questionnaire respondents asserted that their gender identity or appearance was the reason they were targeted. Several women described how the abuse they received tended to emphasize their identity, including references to sexualized acts and derogatory remarks about their professional capabilities based on their gender. One woman explained how she inadvertently put her “whole self” online for scrutiny and, consequently, received many messages laced with “fatphobia and body shaming” (White woman, health journalist). She further explained, “It feels pretty vulnerable to be attacked that way as a young woman... I think it’s really been probably the most intense misogyny I’ve ever faced in my life.”

**Figure 1:**
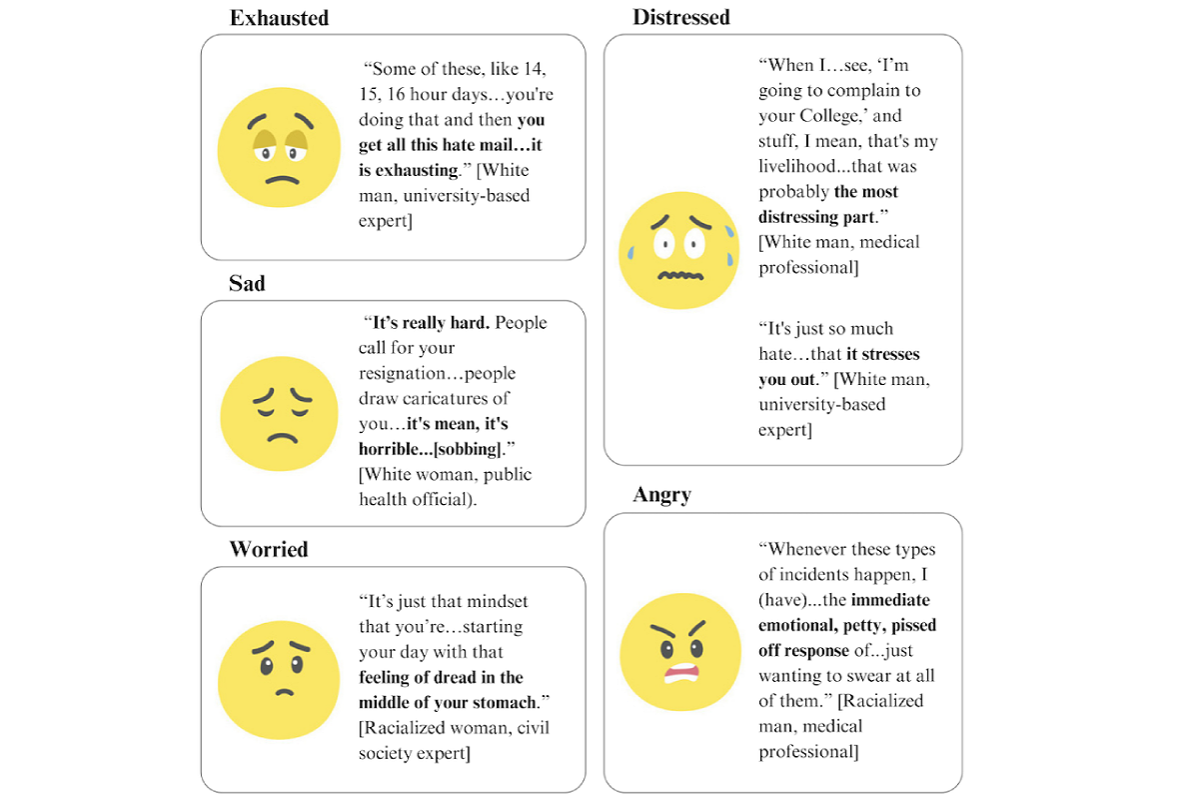
Examples of negative emotions expressed by interviewees because of online abuse.

When interviewees recounted the online harassment they experienced, they often described negative emotions (Figure 1). Similarly, 41% (14/34) of the questionnaire respondents experienced strong negative feelings in response to online harassment (Table 2).

Most health communicators mentioned multiple negative emotions around online abuse. Interviewees commonly mentioned feeling simultaneously frustrated and exhausted when they received a “constant barrage” of online harassment (White woman, public health official). Health communicators were “being fed this stream of negativity and abuse through...your phone all day” (White man, health journalist), and some felt that “even the best possible...person can’t deal with an assault of hostility 24-7” (White woman, public health official).

An interviewee reflected on how online harassers were misrepresenting his opinion on certain health topics:

That really does sting, and you find it frustrating and you waste...cognitive energy worrying about it.

White man, university-based expert

Several health communicators explicitly mentioned the “psychological toll” and “psychological exhaustion” of receiving and reading countless negative, hostile, and threatening e-mails (White man, university-based expert).

Interviewees also frequently expressed feeling sad and scared in the same instance:

When I get a hateful message, a...message that threatens violence against me, it makes me feel sad. ...it does scare me. It instills fear in me, and...it makes me feel really sad to know that this person...has taken time out of their day to...send me that message with the hope of hurting me somehow.

Racialized woman, civil society expert

While some of the interviewees described symptoms of depression, such as “not wanting to get out of bed...[in the] morning” (Racialized woman, civil society expert), others described symptoms of anxiety, such as difficulty “trying to unplug” (White woman, health journalist). Another interviewee, who received an email with “a message from someone...saying they hope that I get blood clots and like, basically die,” explained how these types of messages are anxiety-inducing: “sometimes it keeps you up at night, makes you very worried and concerned” (White woman, health journalist).

Racialized and women health communicators across interviews and workshops discussed how negative comments about their ethnicity, ancestry, and physical appearance impacted their mental health and well-being:

When it actually ends up being personal attacks on you as a person, on how you look, on the colour of your skin, where you’re originally from...It takes a toll on you.

Racialized woman, civil society expert

Interestingly, only 18% (6/34) of the questionnaire respondents claimed to be “struggling with mental health issues” (Table 2), whereas the interviewees repeatedly mentioned how their mental health and well-being had been impacted by health communication and subsequent harassment during the pandemic. This variation in the severity of mental health impacts reported suggests that certain participants implemented strategies to mitigate some of the mental health consequences of online abuse.

**Table 2:** Impacts of online harassment on questionnaire respondents.

		Questionnaire respondents (N=34), n (%)
**Personal impacts**		
	Experienced strong negative feelings (eg, fear, horror, anger, guilt, or shame)	14 (41)
	Felt scared for the safety of their family and friends	8 (24)
	Felt scared for their physical safety	7 (21)
	Struggled with mental health issues	6 (18)
	Felt jumpy or easily startled	6 (18)
	Experienced strong negative beliefs about themselves or other people	6 (18)
	Repeated, disturbing dreams of the stressful experience	3 (9)
**Professional impacts**		
	Avoided publicly addressing certain topics	21 (62)
	Seriously considered quitting health communication	14 (41)
	Requested a change in their professional role	5 (15)
	Took a greater number of sick days than usual	2 (6)

### Coping Strategies Implemented by Health Communicators

**Figure 2:**
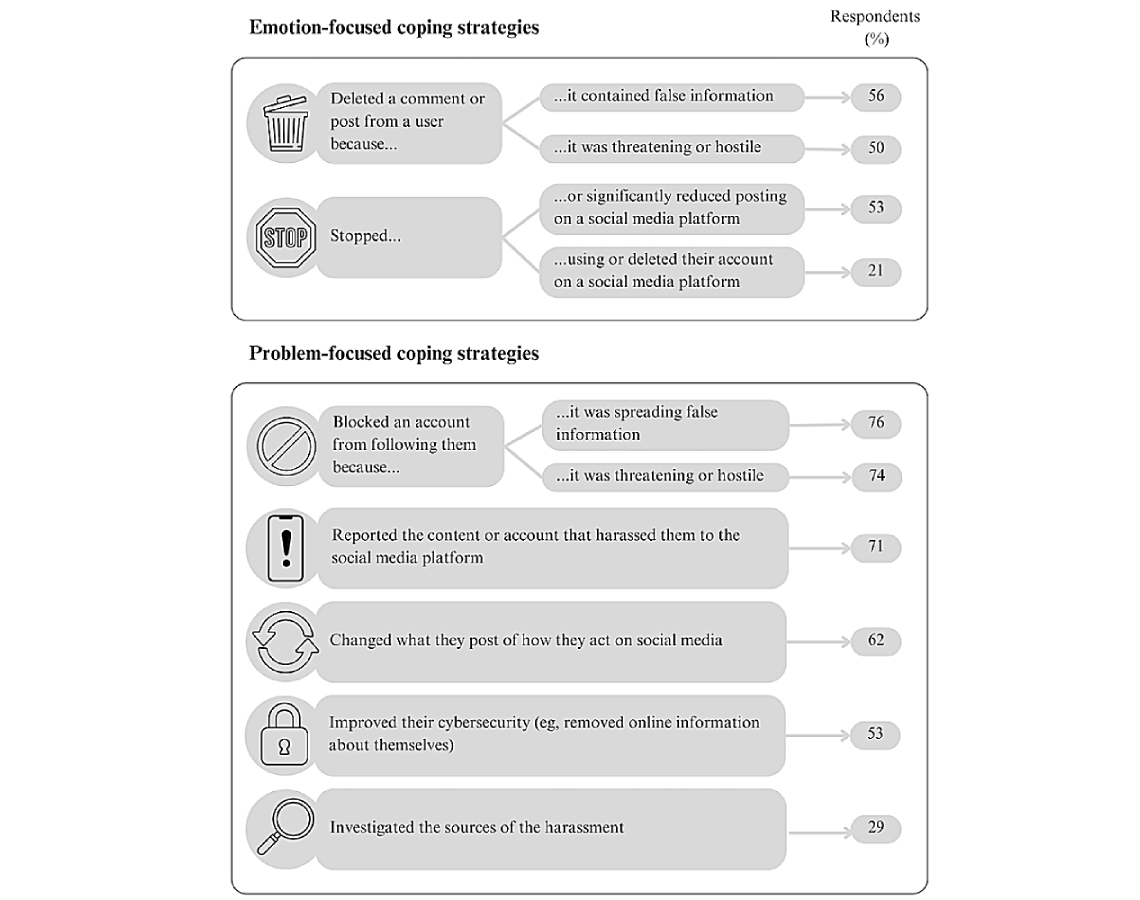
Responses to online harassment implemented by questionnaire respondents (N=34).

When health communicators experienced online harassment, they used a variety of coping strategies (Figure 2).

#### Emotion-Focused Coping Strategies

Health communicators’ most common emotion-focused coping strategies were (1) withdrawing from social media planforms, (2) avoiding social media platforms altogether, and (3) accepting online harassment and abuse as unavoidable.

First, many participants withdrew from online public health communication because of harassment. In total, 53% (18/34) of questionnaire respondents noted that they significantly reduced or stopped posting on a social media platform (Figure 2). Interviewees mentioned that they “used to...be very active on Twitter,” (White woman, health journalist) but they “made a conscious choice to distance myself from Twitter as a professional” due to persistent online harassment (White man, health journalist).

Second, 21% (7/34) of questionnaire respondents claimed that they have stopped using or deleted their account on a social media platform (Figure 2). Several health communicators explicitly stated that they avoided social media platforms to safeguard their mental health and well-being. “I felt for my mental well-being, I am avoiding this because it is like a triggering response to see all those notifications coming in again and again and again” (White woman, health journalist). One interviewee said he had “to turn if off” for his “own peace of mind...because there’s been an overwhelming amount of negativity,” (White man, public health official), and another firmly stated she was “not on Twitter for...[her] mental health” (White woman, public health official).

Several interviewees justified these emotion-focused coping strategies by explaining how sharing public health information was not part of their job descriptions, so they were “not paid to be on Twitter” (Racialized man, medical professional)*.* A racialized civil society expert noted, “It’s not as though this is my job...I’m not getting paid for any of this work...[and] on top of that, I get vitriol.”

Third, several interviewees seemed to accept the possibility of receiving online harassment. One health communicator conceded that “because of the way that things are now, whatever you share, anything you post...you do open yourself up to some degree of abuse” (White man, health journalist). Another felt disheartened that she was “going to have to be dealing with [online harassment]...for a long time,” particularly if she wanted to continue publishing in major outlets and working on “more polarizing” topics (White woman, health journalist).

Health communicators may have relied on emotion-focused coping strategies because they were unaware of problem-focused coping strategies or unable to implement them. One woman asserted that she did not “know how to get back on Twitter without...facing all this same garbage again” (Health journalist). Another admitted that she felt “ill-equipped to...be on the site anymore” (White woman, health journalist).

#### Problem-Focused Coping Strategies

Scarduzio et al [21] proposed 5 specific “types” of problem-focused coping strategies, including blocking or unfriending, changing online behavior, formal help-seeking, peer intervention, and confronting. Health communicators in our study implemented all 5 of these digital coping strategies to mitigate further online harassment and abuse.

In total, 74% (25/34) of the questionnaire respondents blocked a threatening or hostile account from following them, and 53% (18/34) of respondents improved their cybersecurity (Figure 2). Several interviewees explained the exact steps they took to improve their cybersecurity, such as “taking my face off of my Twitter profile” (White man, health journalist)*.* One interviewee explained how making her “account private” helped her create “a bit of a safer space” online (Racialized woman, civil society expert).

In total, 62% (21/34) of the questionnaire respondents (Figure 2) and many interviewees changed what they posted and how they behaved online. One participant started making an extremely conscious effort to refrain from posting personal information online:

It has just made me more cognizant of what I share in general about my life...I never tweet about where I am. I never tweet about the neighbourhood I live in...I didn’t want malicious people [to] have that information about me.

White woman, health journalist

Other health communicators refrained from sharing public health information because they were “worried about it becoming somehow a lightning rod for hate or harassment or just unwanted negative attention” (White woman, health journalist). For example, some interviewees explained how they no longer shared opinions on “controversial and important topics” (White woman, health journalist), and they “don’t advocate outwardly for...[COVID-19] restrictions” (Racialized man, medical professional). Similarly, 62% (21/34) of the questionnaire respondents avoided publicly addressing certain topics (Table 2). As more health communicators resorted to self-censorship, crucial health information became less available to publics.

Other participants described setting boundaries on how they used social media platforms, not as a form of self-censorship but to balance personal well-being with professional efficacy. For instance, when online abuse made his social media engagements particularly stressful, a health official took time away from the platform to develop new strategies for how he would use it:

I just reactivated [my account] after a few weeks...Coming back with a few rules in mind, I felt much better.

Racialized man, public health official

Some health communicators sought assistance to manage online abuse by reporting the harassment directly via social networking sites’ reporting mechanisms. In our study, 71% (24/34) of questionnaire respondents engaged in “formal help-seeking” by reporting the content or account that harassed them to the social media platform (Figure 2). Several interviewees stated that this action did not have a reliable effect, since in some situations the posts or accounts remained on platforms long after the health communicator had reported them. Our interviewees did not discuss how inconsistent action by social media platforms shaped their assessment of the efficacy of this problem-focused coping strategy.

In total, 41% (14/34) of the questionnaire respondents reported acts of online harassment to a supervisor or employer, and 18% (6/34) of respondents sought help from a supervisor or employer (Table 3). Two health journalists found support and directives from their employers to be effective. One journalist explained how the independent news website she writes for “has been really supportive and proactive” by clarifying “the conditions that my work is expected to continue under” (White woman, health journalist)*.* The other journalist described how the mental health supports that her employers have provided were beneficial:

Both organizations that I’ve worked for have really been putting an emphasis on...getting mental health support. I think some big changes that were made structurally to benefits that were being offered in terms of how much mental health support was being covered made a really big difference.

Racialized woman, health journalist

These findings suggest that health journalists received more support from their employers than health communicators in other professions.

Other health communicators relied on colleagues and family members to help manage online abuse and stop harassers (ie, “peer intervention”). In response to online harassment, more than half of the questionnaire respondents (18/34, 53%) asked a colleague for help, and 47% (16/34) of respondents asked a friend or a family member for help (Table 3). For instance, members of a public health agency team regularly checked in with each other to provide emotional support, often in the form of humor, and to review potentially abusive messages.

If someone else, such as an employee, colleague, or significant other, could oversee their social media accounts, then health communicators would not have to read negative comments and messages themselves:

I was...telling with my campaign team that I actually want to hand...over the keys [to my social media accounts]...because I actually don’t want to see it anymore.

Racialized man, university-based expert

One public health official described how her “partner joined Twitter partly because he took on the job [of] monitoring my account,” but she acknowledged “there’s a toll... when you read angry tweets about your partner every day” (White woman, public health official).

Confronting online harassers was not a popular problem-focused coping strategy used by health communicators. Only one interviewee explained that she wanted to engage with perpetrators of online harassment “because criticism and conflict eats at” her, and it was “empowering” to try to connect with those who were unnecessarily hostile (White woman, university-based expert). She noted that, “I’ve engaged twice, by phone and it actually worked.”

**Table 3:** Sources of reporting and support for questionnaire respondents who experienced online harassment.

		Questionnaire respondents (N=34), n (%)
**To whom did you report the acts of online harassment?**		
	Social media platforms	22 (65)
	Supervisor or employer	14 (41)
	Professional association or governing body	6 (18)
	Police	4 (12)
	Government or political representative	1 (3)
	Unions	1 (3)
	I did not report any acts of harassment	5 (15)
**From whom did you seek support?**		
	Asked a colleague for help	18 (53)
	Asked a friend or family member for help	16 (47)
	Spoken publicly about the experience of being harassed, having your reputation attacked, or the sources of harassment	13 (38)
	Looked for online resources to protect yourself or cope with harassment	9 (26)
	Asked my supervisor, employer, or organization for help	6 (18)
	Sought help from a professional organization or other civil society group	5 (15)
	Sought legal advice	5 (15)
	Sought medical or psychological help	4 (12)
	I did not look for support	3 (9)

### Continuation of Online Health Communication

Health communicators’ experiences of online abuse prompted them to reflect on their long-term use of social media to engage publics. We identified 2 broad groups: those who felt the negative impacts of online harassment on their mental health and well-being outweighed the potential benefits of public health advocacy and those who expressed a desire to continue sharing relevant public health information despite online harassment. Health communicators discussed the internal conflict between reducing their engagement to protect their mental health and continuing their advocacy. One interviewee, after being targeted on Twitter for discussing abuse she and other journalists faced, stopped engaging with those issues online:

That was honestly really frustrating because I felt that at a time that I really needed to be vocal about these things, I couldn’t without compromising my safety and my mental well-being. It kind of felt like there was no good option: either stay silent about what had been done or speak out and perhaps welcome more harm.

White woman, health journalist

Those in the first group generally reduced their online engagement to prioritize their mental health and well-being:

My mental health and well-being are more important than the hope that maybe...these people will learn...because they’re not going to learn.

Racialized man, medical professional

Similarly, a participant who used online platforms to remain engaged with health issues reported that he began to believe those benefits are being outweighed by “the risks to my mental health and well-being...and the threats to my productivity” (Racialized man, university-based expert).

In total, 41% (14/34) of questionnaire respondents seriously considered quitting health communication (Table 2). An interviewee explained how burnout prompted him to take a step back from his profession during the pandemic:

I was just kind of tired, I guess...I certainly felt burnt out from reporting on the pandemic...There are still COVID stories that are important to tell and there’s important journalism to be done, but I felt as if I didn’t want it to be done by me anymore.

White man, health journalist

Conversely, interviewees in the second group explained why and how they would continue to engage in online health communication despite the challenges:

I do feel upset for a little bit (after experiencing online abuse), but it has never gotten to a point where I would think to myself, “I’m not going to do this ever again.” I know of people who have given up, who’ve taken time off or just completely stopped engaging, but...at least for now, I haven’t reached that point.

Racialized woman, civil society expert

Several participants emphasized that they continued to advocate online because they believed in the public benefit of sharing health information:

I’m just trying to tell (people) the facts. And to be targeted for being the messenger of those facts is not very fun. There have been times because of the backlash that I’ve thought, well, maybe I won’t tweet as much. And I definitely had that thought a few times during the course of the pandemic. I really had to weigh...(is) me getting a few messages that are annoying more important than me trying to get information out to people? And for me, getting information out is always more important.

Racialized woman, health journalist

Although most health communicators implemented a variety of problem-focused and emotion-focused coping strategies (Figure 2), only some demonstrated a strong willingness to continue their online engagement, whereas others contemplated quitting public health communication. In the face of persistent online abuse, *continuing* to post online could be understood as an act of “professional resilience.” Health communicators who faced challenges overcoming the mental health and well-being impacts of online abuse were more likely to reduce or abandon their online advocacy efforts.

## Discussion

### Impacts of Online Abuse

Throughout the COVID-19 pandemic, many health care providers, researchers, public health officials, and health journalists put extraordinary effort into engaging publics online, which often exposed them to unwanted harassment and abuse [10]. Although online harassment is often dismissed because it occurs in virtual environments, the consequences of such harassment can be very real, including psychological stress and burnout [13]. Among our questionnaire respondents, 82% (28/34) faced online harassment or abuse. Therefore, most participants reported negative emotions, including feeling fatigued, sad, distressed, and angry. Some participants shared symptoms of anxiety and depression, and some explicitly reported that they had been struggling with mental health issues. This emotional distress caused by online harassment has exacerbated the widespread burnout experienced by medical professionals during the COVID-19 pandemic [26].

### Coping Strategies for Online Abuse

Participants in this study mentioned a variety of strategies to cope with the mental health impacts of online harassment. Drawing on the framework proposed by Lazarus and Folkman [17], we categorized these as emotion-focused and problem-focused coping strategies [21,27,28].

Participants’ most common emotion-focused coping strategies were enduring or ignoring online harassment and disengaging or withdrawing from social media platforms. Several interviewees seemed to accept online harassment as something that came with the territory, rather than something that could be mitigated with problem-focused coping strategies. This sentiment aligns with other research findings that many scientists who publicly commented on the COVID-19 pandemic said they learned to cope with online harassment by “accepting it as an unpleasant but expected side effect of getting information to the public” [10]. Furthermore, research suggests that many health communicators have purposefully ignored and not responded to social media trolls [10,11,29], since “engagement is their oxygen” [5]. Other emotion-focused coping strategies for online abuse reported by health communicators in our study and the literature include deleting negative comments, reducing engagement on social media platforms, avoiding certain social media platforms, and deleting social media accounts altogether [5,10,29]. Although self-blame has been identified as an emotion-focused coping strategy in other studies [16], no health communicator in our study described blaming themself for the online harassment they received.

Health communicators also used many problem-focused coping strategies to respond to online abuse. As opposed to reactive emotion-focused strategies, such as deleting negative comments, problem-focused strategies tend to be more proactive, such as blocking or reporting hostile users. For example, by preventing such users from sending messages directly to the communicator or seeking to have social media platforms enforce their terms of service against online harassment, health communicators have tried to limit the number of negative or threatening messages they will receive *in the future*. One academic told other researchers that she even blocked her abuser’s followers to make it harder for them to target her [10]. Health communicators in our study and in the literature have taken several proactive steps to avoid receiving online abuse, including refining cybersecurity settings by making accounts private [11,29] and removing contact information from public websites [5].

Furthermore, rather than avoiding posting on social media platforms *entirely*, many health advocates became “more careful about how...[they] use” social media [10], making conscious efforts to strategically avoid posting about *specific* topics online. In fact, 63.5% (228/359) of the physicians, biomedical scientists, and trainees in the United States who reported experiencing any online harassment during the pandemic claimed that they have *changed* how they use social media [12]. Many health communicators, in the literature and our study, have begun compartmentalizing professional and personal identities online, avoiding “making comments that might be perceived as political” or controversial [10], or refusing to correct misinformation online [29].

When we examined help-seeking behaviors among our participants, we found a distinction between *reporting* online harassment and *seeking support* for such harassment. While almost three-quarters of questionnaire respondents (24/34, 71%) formally reported online harassment directly to social media platforms, when the same respondents recorded who they asked for help, colleagues (18/34, 53%) and friends and family members (16/34, 47%) were the most common sources of support. Several interviewees explained how their employees or loved ones helped them manage online abuse, limiting the number of negative comments and direct messages they read about themselves. Similarly, Hodson et al [30] reported that women scholars who experienced online harassment were most likely to try to deal with the problem by enlisting the help of spouses, close family members, or friends to help manage their online presence. Social media platforms allow users to report and block hostile users [31], but taking these actions may not be as effective in improving health communicators’ mental health and well-being as receiving assistance and emotional support from friends and family members [30]. However, our study focused on health communicators’ perspectives on the efficiency of various coping strategies, rather than examining social media platform activity. Thus, we cannot directly assess whether health communicators’ reports of hostile users were acted on by platforms or whether these platform responses shaped health communicators’ assessments on the efficacy of this strategy.

Some scholars have discussed how emotion- and problem-focused coping strategies can be difficult to discern, and we also found some overlap between the 2 categories. For example, Scarduzio et al [21] described asking friends and family members “for support and advice” as an active emotion-focused coping strategy yet asking friends and family members “to help stop the harasser” as a problem-focused coping strategy. Thus, we acknowledge that some coping strategies may fall into a “gray zone” between emotion-focused and problem-focused.

Although confrontation was an unpopular problem-focused coping strategy among health communicators in our study, some participants expressed a desire to have productive dialogues with their harassers. One physician said she occasionally responded to comments or messages but not when she was upset or angry [10]. However, confronting perpetrators may pose a risk of further abuse and negative mental health consequences [32], which could be one reason most health communicators in our study and the literature relied on other coping strategies.

We found some problem-focused coping strategies were individual in nature, while other strategies involved support through personal and professional relationships or official organizational policies. Individual strategies, like blocking hostile accounts, can lessen exposure to online abuse and, consequently, lessen the impacts of such abuse on mental health and well-being. Another strategy to lessen exposure is sharing the burden of monitoring and responding to hostile content with friends, loved ones, or colleagues. Beyond reducing exposure to online abuse, social and organizational support can strengthen a health communicator’s ability to emotionally process abuse and rebuild mental health. For example, 2 journalists in our study, who demonstrated a willingness to continue their professional advocacy, highlighted the importance of access to expanded employee benefits, which enabled them to take time off work and receive counseling after experiencing online abuse. In the workshops, several public health officials described how their teams routinely discussed the hostility they received to address any sense of isolation or personal responsibility for these reactions and to share coping strategies. Conversely, health communicators in our study who worked as freelancers or in individual medical practices noted that a sense of isolation and lack of workplace support had exacerbated the mental health consequences of online abuse.

### Professional Resilience Among Health Communicators

There are several opinions about the effectiveness of emotion- and problem-focused coping strategies, but many scholars argue that problem-focused strategies are more beneficial in the long-term [17,33]. Our findings suggest that health communicators who used problem-focused coping strategies were more likely to continue their advocacy than health communicators who used emotion-focused coping strategies. In the face of persistent online harassment and abuse, Veletsianos et al [16] reported that women scholars who continued working and teaching “required determination and resilience.” Thus, simply continuing their professional obligations became an act of resistance [16]. Similarly, we put forth that health communicators who remained “active” online demonstrated significant professional resilience, compared to those who censored or otherwise minimized their online presence. Several communicators in our study noted their commitment to sharing important health information broadly with publics was one reason for this resilience. Some of the clearest expressions of professional resilience were shared by racialized health communicators in our study, which warrants further investigation.

Importantly, we do not define professional resilience as an individual quality or character trait. An individual’s capacity to continue using online spaces to inform and advocate publics is significantly shaped by the forms and intensity of online abuse they face as well as the interpersonal and institutional support they receive. Moreover, the extraordinarily high levels of online engagement by health communicators during the COVID-19 pandemic required many health experts to take on burdens that went beyond their job descriptions or were otherwise unsustainable.

Online and in-person abuse has contributed to burnout and high turnover among health communicators, particularly medical professionals and health journalists [11]. Many participants in our study contemplated reducing or ceasing their online health communication activities. This decision could have negative professional consequences, such as a reduction in opportunities to network and collaborate with other scholars [9]. These consequences were especially pronounced for women and racialized individuals, who have historically been excluded from academia. Women who have reported considerable online harassment, especially sexual harassment, have frequently responded by reducing and censoring their online participation as well as deleting their accounts on social media platforms [9,16,34], further limiting their opportunities for professional development.

There are also broader social consequences if health communicators reduce their engagement online. Notably, misinformation and disinformation may be left unchecked by those most qualified to counter it [12]. Experts who were attacked online said they were less likely to participate in future media interviews, highlighting the effectiveness of these attacks [7]. Similarly, scientists who reported the highest frequency of trolling in the *Nature* survey were most likely to report that their experiences have greatly affected their willingness to speak to the media in the future [10]. At a time when “we’ve never needed them so badly” [10], many health communicators are avoiding certain topics on social media or withdrawing from these platforms entirely. Furthermore, given the alarming amount of abuse reported by senior public health officials, it seems likely that the hostile online environment could dissuade up-and-coming health communicators from fully engaging in important discussions [6]. Consequently, we may see a reduction in the diversity of thoughts and opinions shared within academia and public discourse, especially if women and racialized academics are disproportionately pushed out of online spaces [16].

### Policy Recommendations

Health communicators in our study implemented various emotion- and problem-focused coping strategies, many of which they implemented as individuals. Future studies should investigate the effectiveness of these digital coping strategies for health communicators’ mental health, well-being, professional efficacy, and professional resilience, especially those who belong to gender and racial minorities.

Our findings also highlight the limitations of individual coping strategies, necessitating the development of organizational policies to support those who receive online abuse and sanction those who perpetrate it. While health communicators have taken many steps to mitigate the frequency and severity of harassment they experience on social media platforms, advocates argue that individuals should not have to “cope on their own” [10].

Advocates have asserted that there is much that institutions can do to assist scientists who are receiving online abuse [10]. Studies have suggested several actions for institutions that employ health communicators: creating formal policies to guide health communicators’ digital interactions [19], hosting trainings for health communicators on how to engage with the media and what to expect from online trolls [5,10], and enlisting organizations’ information technology departments to block consistent abusive emailers and report incidents to social media platforms and police [10]. These organizational efforts should address the potential for different forms of abuse based on individuals’ gender identity, race or ethnicity, ancestry, and other sociodemographic characteristics. In our study, the strongest examples of organizational policies were provided by health journalists and communicators at public health agencies. These examples include (1) clear recognition from superiors that online abuse is a serious problem that requires action and (2) institutional programs providing psychological therapy, cybersecurity assistance, and peer support. This finding warrants an exploration of organizational polices across industries to ascertain and promote best practices. Programs are also needed to support health communicators who are not full-time employees of large organizations, such as family doctors, free-lance journalists, and others.

While individual actions may have immediate short-term outcomes, institutional policies and practices could have sustained long-term outcomes for health communicators’ mental health and well-being by preventing online harassment or, at least, mitigating it. Organizational policies would support professional resilience, ensuring that important health information is “not silenced” by online abuse and can still reach publics [10].

### Limitations

There are several limitations to this study. Our purposive sampling of health communicators who were highly engaged in online health communication provided findings from an important population but not necessarily a representative one. Our study was conducted in English, which might have precluded insights from Canada’s significant French-speaking population. Moreover, because of the small sample size of our study, we could not quantitatively compare exposure to online harassment by gender identity, ethnicity, or professional role. A more comprehensive sample of health communicators across institution types, professional roles, and sociodemographic characteristics could identify broader patterns and gaps in our findings as well as greater insights into the experiences and coping strategies of members from marginalized populations. Finally, this study focuses on health communicators in Canada and their experiences during the first few years of the COVID-19 pandemic, when uncertainty and fear were heightened. Further comparative studies across countries are needed to measure the long-term impacts of online abuse and coping strategies on health communicators in different political and health care contexts.

### Conclusions

This study elucidates the significant impacts of online abuse on health communicators during the COVID-19 pandemic, highlighting both mental health and professional consequences. Despite the variety of individual coping strategies used by health communicators, there remains a pressing need for organizational efforts that offer comprehensive protection against online abuse and support for those who receive it. Institutions must acknowledge that the burden of coping with online abuse should not fall solely on individuals and that they should be supported by formal organizational policies and practices that safeguard the mental health, well-being, and professional efficacy of health communicators. These efforts will support individuals at the forefront of public health communication to share critical information.
